# Gamma-Ray Treatment of* Echinococcus* Protoscoleces prior to Implantation in Mice Reduces Echinococcosis

**DOI:** 10.1155/2016/9027489

**Published:** 2016-08-16

**Authors:** Qing Yuan, Bo Li, Shiping Jiang, Qiang Zhao, Ji Duo, Xiang Huang

**Affiliations:** ^1^Department of General Surgery, Ya'an People's Hospital, Ya'an, China; ^2^Hepatobiliary Surgery, The Affiliated Hospital of Luzhou Medical College, Luzhou, China; ^3^Ganzi State Hospital, Kangding, China

## Abstract

Echinococcosis is a serious parasitic disease caused by* Echinococcus* tapeworms. Protoscoleces are sometimes released during surgical treatment for hydatid cysts, causing the recurrence of echinococcosis. Protoscoleces may be susceptible to radiation therapy. In this study* Echinococcus* protoscoleces were cultured* in vitro* and then divided into four different *γ*-ray irradiation dose groups (10 Gy, 20 Gy, 40 Gy, and 80 Gy) and a blank group. The protoscoleces were then implanted into the abdominal cavity of mice. Four months later, we observed that the incidence and weight of cysts declined with the increase of irradiation dose. *γ*-ray irradiation can suppress the generation of* Echinococcus* originated from protoscolex, the reason of which is due to the damaging to the structure of* Echinococcus*. Irradiation may prevent echinococcosis recurrence after surgical removal of hydatid cysts.

## 1. Introduction

Echinococcosis or hydatidosis is a parasitic disease in humans and animals that may cause serious problems. This type of zoonosis is characterized by growth of hydatid cysts in hosts. The two major infective species in humans are* Echinococcus granulosus* and* E. multilocularis*, which cause cystic echinococcosis and alveolar echinococcosis [1]. In addition* E. vogeli* and* E. oligarthrus* cause polycystic echinococcosis [2]. It is thought that 58% of the total population in Central Asia are at risk of cystic echinococcosis and surgical incidence for echinococcosis is as high as 10% in some Tibetan communities in western China [3]. Alveolar echinococcosis is also endemic in Central Asia with up to 6% of some village communities infected [3]. The major infective site for the two most common forms is the liver and occurs in approximately 75% of cases [2]. Hepatic echinococcosis can involve hepatic enlargement, epigastric pain, nausea, and vomiting. The sudden rupture of a cyst can cause allergic reactions including fatal anaphylaxis. The lungs are also commonly infected; ruptured cyst membranes in the lungs can sometimes be retained and act as a site for bacterial or fungal infection [4].

Prevention of infection is an important basis for control of echinococcosis; monthly deworming of dogs with praziquantel, as a key measure to control the* Echinococcus* parasites, has been used in western China while education and vaccination of livestock are also important [3]. Once infection has occurred the main treatment options are surgery to remove the cysts and drug therapy with benzimidazole compounds as well as other methods such as cyst puncture, aspiration, injection of chemicals, and reaspiration [4]. For* E. granulosus* the* Echinococcus* protoscolex is an important part of the tapeworm's life cycle and a central link for onset of hydatidosis in humans; protoscoleces attach to the intestinal mucosa of the dog and develop into the adult tapeworm after 30 to 80 days [2]. Since no drugs can effectively kill a protoscolex [5], it is an important factor that also affects the prognosis of surgical treatment [6, 7]. Implantation of protoscoleces caused by operative egress of hydatid cyst fluids during surgery is a serious issue for recurrence [8]. So an effective mechanism of killing the protoscoleces during surgery should assist with the treatment of echinococcosis by preventing recurrence.

Radiation therapy has been considered as a method of treating alveolar echinococcosis that cannot be treated surgically [9, 10]. But the benefits of this method remain under debate [11]. The ionizing radiation of *γ*-rays can cause DNA damage, leading to structural and functional changes to DNA and metabolic changes in cells and in some circumstances cell death [12]. Radiation therapy with *γ*-rays is commonly used as a therapy in cancer to kill tumor cells [13]. An alternative method of using the killing potential of radiation therapy is to treat protoscoleces that may be released during surgery, in a similar way to the drug therapy during surgery and thus help prevent recurrence of the infection. Previous experimental results show that *γ*-ray irradiation kills protoscoleces* in vitro* [14, 15] but is less effective at killing protoscoleces* in vivo* [15].

The aim of this investigation was to evaluate whether *γ*-ray irradiation at different doses would effectively prevent* Echinococcus* protoscoleces from infecting mice. This information should provide details on whether radiation therapy could be used during surgery to prevent recurrence of echinococcosis. By simulation of *γ*-ray irradiation of* E. granulosus* cyst fluid during intraoperative spillover, we found that radiation treatment of protoscoleces decreased the incidence of echinococcosis and the weight of the resulting echinococci, while increasing the level of calcification. This study suggests that radiation therapy with *γ*-rays may be a useful method of reducing the recurrence of echinococcosis by killing protoscoleces in cyst spillover.

## 2. Materials and Methods

### 2.1. *Echinococcus* Protoscoleces

*Echinococcus granulosus* protoscoleces were collected from a hydatid liver surgery in Sichuan Provincial People's Hospital (China). External capsule complete resection was performed and the specimen was maintained at lower than 4°C in an ice box and sent to the laboratory. The harvested liver hydatid cyst was placed on a laminar flow cabinet and its surface was disinfected with 75% alcohol. Cyst fluid was extracted with a 50 mL syringe, with repeated filling and emptying to ensure that the protoscoleces that had adhered to the endocyst were completely collected. After the cyst fluid was completely extracted, the sac wall was cut open, and the surface of the internal capsule was rinsed using physiological saline. The flushing fluid and the hydatid cyst fluid were collected and reserved. Then the sample was centrifuged at 3000 r/min for 5 minutes to obtain the yellow-white hydatid sands. After removing the supernatant, the hydatid sands were rinsed with flushing fluid (physiological saline containing 1500 *μ*/mL penicillin, 1000 *μ*/mL streptomycin) (penicillin-streptomycin solution: ThermoFisher Biochemicals (Beijing), Co., Ltd., China) 3 times. 1 mL of 20% trypsin solution (ThermoFisher Biochemicals (Beijing), Co., Ltd.) was added to the hydatid sands and left for 20 mins to digest the tissue debris. The hydatid sands were then again rinsed with flushing fluid and centrifuged, the supernatant was removed, and finally the protoscoleces were obtained and reserved.

### 2.2. Animals

Specific-pathogen-free level female Kunming mice (*n* = 75), 6 weeks old and mean weight of 20 ± 4 g, were provided by the Animal Laboratory of Luzhou Medical College (Sichuan, China). The mice were housed at 20–25°C and 50 ± 5% humidity with* ad libitum* access to food and water and 12 : 12 h light/dark cycle. All procedures and animal experiments were approved by the Animal Care and Use Committee of Luzhou Medical College.

### 2.3. Incubation of Protoscoleces

Ten mL RPMI-1640 medium (ThermoFisher Biochemicals (Beijing) Co., Ltd.) containing 20% fetal bovine serum (ThermoFisher Biochemicals (Beijing) Co., Ltd.) was added to the cuvette in which the protoscoleces were stored. After agitation, 0.2 mL of the solution was placed on a glass slide and the protoscoleces were counted under a BX50 inverted microscope (Olympus, Japan). The count was repeated for 5 times, and the average value was obtained. Then 20% methylene solution (ThermoFisher Biochemicals (Beijing) Co., Ltd.) was added to the collected solution to observe the survival rate of the protoscoleces (live protoscoleces were not dyed, while dead protoscoleces were dyed blue), which was required to be greater than 90% for this study. Finally the protoscoleces were cultured at a density of 2000/mL in RPMI-1640 medium containing 20% fetal bovine serum and incubated at 37°C in a CO_2_ incubator (ThermoFisher Biochemicals (Beijing) Co., Ltd.).

### 2.4. *γ*-Ray Irradiation

The protoscoleces were cultured and allowed to grow and adapt to the media until evagination, and then they were *γ*-ray-irradiated. During the experiments, the medium containing the protoscoleces was evenly divided into 5 groups, of which one was the control group, and the other 4 groups were the experimental groups. The experimental groups were placed into a Cobalt-60 therapy unit (GWXJ80 type 60 Co teleradiation apparatus: Nuclear Power Institute of China) for irradiation. The exposure dose was controlled in a mediate-and-low range after referring to earlier experimental results [14] to avoid invalid implantation of the protoscoleces due to rapid death after the irradiation. The exposure doses of the 4 groups were 10 Gy, 20 Gy, 40 Gy, and 80 Gy, respectively.

### 2.5. Inoculation

After irradiation, the protoscoleces were inoculated into mice that had been fasted for 1 day. The 75 female mice were randomly divided into 5 groups, 15 in each group, and the abdominal puncture inoculation method was adopted. The puncture site was selected from the left lower abdominal inguinal region towards the right upper abdomen. 1 mL protoscolex medium (about 400–600 protoscoleces) was injected, for each mouse. The mice resumed feeding 12 hours after this procedure.

### 2.6. Detection of* Echinococcosis* in Mice

Four months after the implantation of protoscoleces, blood samples were collected from the mouse hearts to detect* Echinococcus* antibodies. To observe the occurrence of false positives, blood was also collected from 10 healthy mice. The detection methods were in accordance with the instruction of the kits (Diagnostic Kit for Antibodies to* Echinococcus*: Guangzhou Jianlun Biotechnology Co., Ltd., China). The absorbance value (optical density, OD) was read on Multiskan FC microplate reader (Thermo Fisher Scientific, USA) at 450 nm as a test wavelength (620 nm as a reference wavelength). According to the kit instructions, the OD of the negative control group should be less than 0.15, and the OD of the positive control group should be greater than 0.5, indicating that the operation is correct and the experiment is valid. The cut-off value (COV) was calculated according to COV = mean OD of negative control × 3.1. So the IgG positive response was defined as sample in the experiment group OD value ≥ COV value, and IgG negative response was defined as sample OD value < COV value.

### 2.7. Development of* Echinococcus*

The mice were killed by cervical dislocation method. After echinococcosis was confirmed by antibodies against* Echinococcus*, abdominal laparotomy was performed. Intra-abdominal cysts were harvested, and after removing the outside tissues, they were dried using clean, dry gauzes and weighed. The suppression capsule rate (suppression capsule rate (%) = [1 − (mean weight of cyst in the experimental group/mean weight of cyst in the control group)] × 100%) and incidence of* Echinococcus* (incidence of* Echinococcus* (%) = number of inoculated mice with hydatid cysts/total number of inoculated mice × 100%) of each group were analyzed.

### 2.8. HE Staining

Hematoxylin and eosin (HE) staining of thin sections of the intra-abdominal cysts was performed according to standard techniques.

### 2.9. Calcium Alizarin Red Staining

Calcium alizarin red staining was performed according to the recommended procedure of the kit (GENMED Paraffin section calcium alizarin red staining kit: Shanghai Jiemei Genetic Pharmaceutical Technology Co., Ltd., China).

### 2.10. Statistical Analysis

All statistical analyses were conducted using SPSS version 17.0 (SPSS Inc., Chicago, IL, USA). Data were expressed as median (quartile) and analyzed by Kruskal-Wallis *H* test. Mann-Whitney *U* was applied to adjust data in pairwise comparisons according to the characteristics of the data. Proportions were analyzed by Chi-square analysis. A *P* value of <0.05 was considered statistically significant.

## 3. Results

### 3.1. Model Development

After abdominal cavity implantation of *γ*-ray irradiated protoscoleces, the mice were in good condition and none of the mice died. Three months after implantation, soft bumps were palpable at the abdomen wall in individual mice.

### 3.2. *Echinococcosis* Rate Estimated by Antibody Detection

By referring to a COV = mean OD of negative control group × 3.1, we obtained the COV = 0.43 for our testing. Our results showed that serum detection was positive in all mice that had incubation of protoscoleces, and serum detection was negative in mice without incubation of protoscoleces.

### 3.3. Visual Observation of Cysts

**Figure 1:**
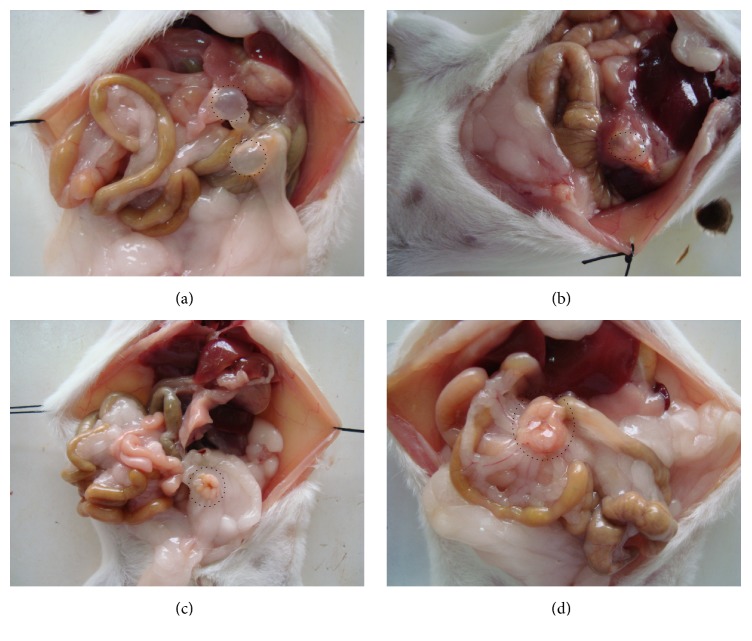
Visual observation of cysts. (a) Rounded hydatid cysts (circled with a dotted line) in the intraperitoneal space of mice in the control group. (b) Rounded hydatid cysts (circled with a dotted line) present the cyst and surrounding tissue dense adhesions in the intraperitoneal space of mice in the 40 Gy group. (c and d) Rounded hydatid cysts (circled with a dotted line) present the degraded morphology in the intraperitoneal space of mice in the 80 Gy group.

**Figure 2:**
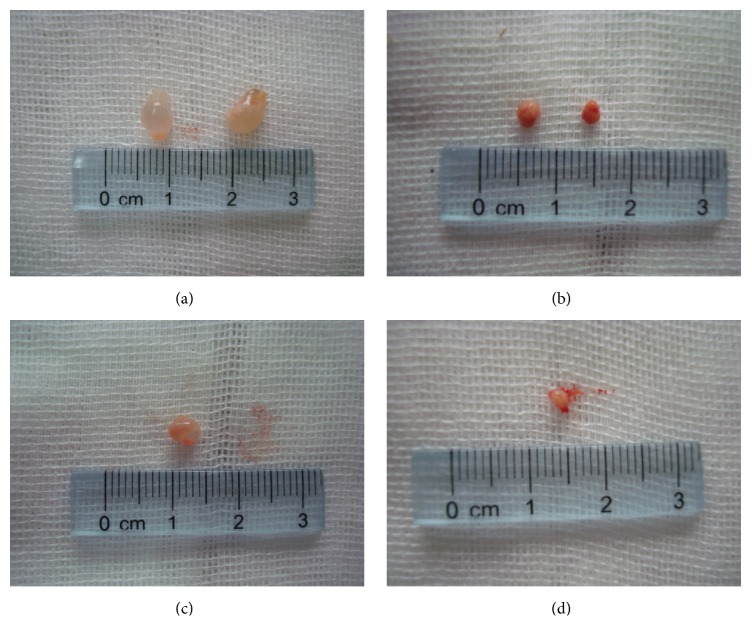
Visual observation of cysts. Control group (a, c)* Echinococcus granulosus* volume is larger, the wall is transparent, and the cystic fluid is clear, compared to the experimental group ((b) at 40 Gy and (d) at 80 Gy) where* Echinococcus granulosus* volume is smaller, the wall is consolidated, and the cystic fluid is turbid.

Cysts were found in the abdominal cavity of the mice, and most of them were located in the abdominal wall and mesentery (Figure 1). Through observation and comparison, we found that the cysts in the abdominal cavity in the control group had a larger volume and were lightly adhesive to the surrounding tissues. The cysts were of rounded shape, white, pale yellow, or transparent, and the cystic wall was elastic and contained clear fluids. On the other hand, the cyst volumes in the experimental groups were relatively smaller and more adhesive to the surrounding tissues. The cystic wall was thickened and some of them presented as a degraded morphology (Figure 2). In some mice in the experimental groups, no cysts were found in the abdominal cavity.

### 3.4. Incidence of* Echinococcus* by Abdominal Cavity Examination

In some mice in the experimental groups, we did not find any cysts in the abdominal cavity. Analyses found that incidence of cysts in each of the experimental groups reduced with increasing radiation dose (*χ* ^2^ = 7.82, *P* = 0.005) (Table 1), indicating that the incidence of cysts is suppressed with increasing exposure dose.

### 3.5. Weight of Isolated Cysts

The median weight of cysts decreased with radiation treatment (*Z* = 32.729; *P* < 0.001), from 157.80 (0.15) mg (median (Quartile)) in the control group to 35.80 (0.07) mg in the 10 Gy group, 0.00 (0.08) mg in the 20 Gy group, 0.00 (0.09) mg in the 40 Gy group, and 0.00 (0.00) mg in the 80 Gy group (Table 2).

In view of the data distribution of the cysts weights in the experimental groups, we used the median weight of each group to calculate the average weight of the hydatid cysts and the suppression capsule rate (Table 2). At 80 Gy, this was 99.61% capsule suppression.

### 3.6. Analysis of Cyst Morphology

**Figure 3:**
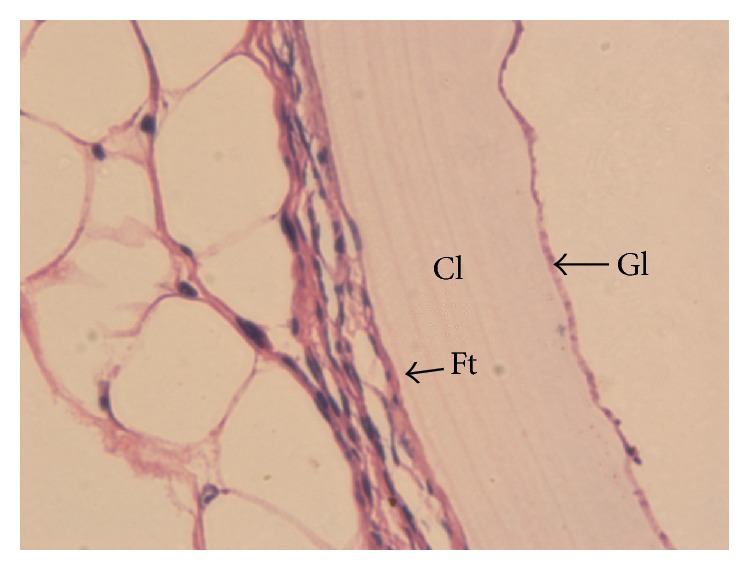
Hematoxylin and eosin (HE) staining to show the structures of the hydatid cysts. HE staining to show the structures of the hydatid cysts typical* Echinococcus* structures that can clearly be seen in the germinal layer (Gl), the cuticle Layer (Cl), and fibrous tissue (Ft) (×200).

In the control group, HE staining of cysts showed that the cyst wall structure was uniform and clear, in which we could observe the germinal layer (Gl), cuticle layer (Cl), and fibrous tissue (Ft). Therefore we confirmed that the cyst was* Echinococcus*, and we did not find any asci or protoscoleces in the Gl (Figure 3).

**Figure 4:**
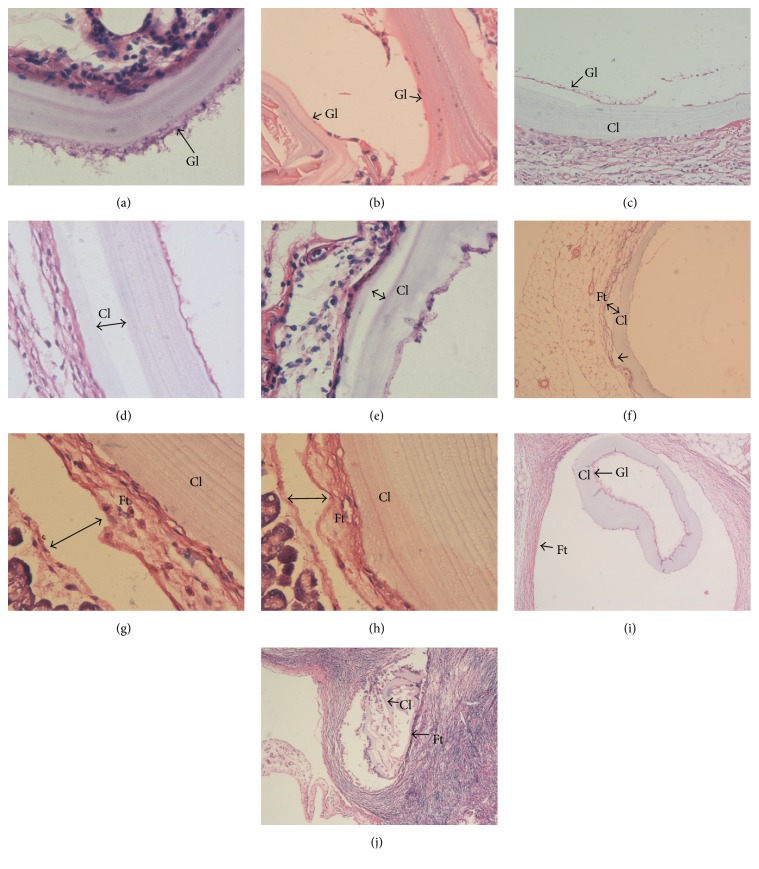
H&E staining to show the structures of the hydatid cysts in the irradiated groups. (a) The hydatid cyst germinal layer (Gl) cells were coarse in the 10 Gy group (×200). (b) The hydatid cyst germinal layer disappeared in the 10 Gy group (×200). (c) The hydatid cyst germinal layer and cuticle separated and formed vacuoles in the 10 Gy group (×200). (d) The hydatid cyst cuticle occurred at separation in the 20 Gy group (×200). (e) The hydatid cyst cuticle occurred at separation in the 20 Gy group (×200). (f) The hydatid cyst cuticle and fiber layer occurred at separation and formed vacuoles in the 40 Gy group (×100). (g)* Echinococcus* fiber layer tissue isolation in the 40 Gy group (×400). (h)* Echinococcus* fiber layer tissue isolation in the 40 Gy group (×400). (i) Hydatid cyst cuticle rupture (×200). (j) Hydatid cyst cuticle rupture in the 80 Gy group (×200).

Comparing the Gl, Cl, and Ft in* Echinococcus* slices from each group showed that, in the control group, the* Echinococcus* cyst wall had uniform thickness. Cells were completely distributed in the Gl with consistent thickness, and no protoscoleces or brood capsules were found. The Cl showed good morphology and was transparent. The Ft was closely textured and tightly adhered to the Cl and tissues (Figure 3). On the contrary, we found a variety of structural damage in the* Echinococcus* cyst walls in the experimental groups (Figure 4). In the 10 Gy group the hydatid cysts' germinal layers were coarse and in some cases had disappeared; the germinal layer and cuticle had separated and formed vacuoles. In the 20 Gy group the hydatid cyst cuticle occurred at separation. In the 40 Gy group the hydatid cyst cuticle and fiber layer occurred at separation and formed vacuoles. In the 40 Gy group the* Echinococcus* fiber layer was isolated. While in the 80 Gy group the hydatid cysts' cuticles ruptured.

### 3.7. Calcification of* Echinococcus*

**Figure 5:**
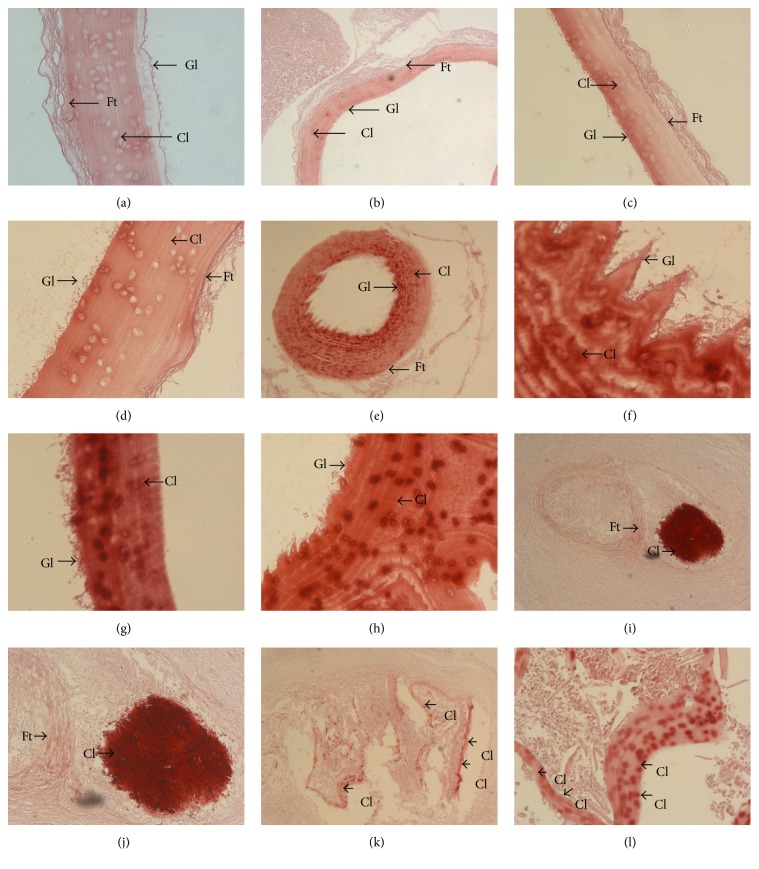
Calcium alizarin red staining of hydatid cysts. (a)* Echinococcus granulosus* stained uniformly and had no calcium deposition in the control group (×200). (b) (×50). (c)* Echinococcus granulosus* with 1/3 of the inner surface of the cuticle appearing to have calcium deposition in the 10 Gy group (×200). (d) (×400). (e) Whole* Echinococcus granulosus* cuticles had calcium salt deposits in the 20 Gy group (×50). (f) 20 Gy group hydatid cyst cuticle layer had calcium deposition, while inside the cuticle showed wave-like erosion (×400). (g) 40 Gy group* Echinococcus granulosus* cuticles had calcium salt deposits (×200). (h) 40 Gy group hydatid cyst layers had calcium deposition (×400). (i) 80 Gy group hydatid cyst cuticles collapsed and formed calcified lesions (×100). (j)* Echinococcus* cuticles collapsed and showed calcification in the 80 Gy group (×400). (k) Hydatid cyst cuticle fracture in the 80 Gy group (×50). (l) (×200). Gl: germinal layer; Cl: cuticle layer; and Ft: fibrous tissue.

Alizarin red staining was uniform without calcium staining phenomenon in the control group. Meanwhile, we found intermittent calcareous infarct phenomenon in the CI in the experimental groups, and this phenomenon becomes more significant with increasing exposure dose (Figure 5). In the 10 Gy group 1/3 of the inner surface of the cuticle had apparent calcium deposits, in the 20 Gy group the whole cuticle had calcium deposits and inside the cuticle appeared wave-like, in the 40 Gy group the cuticle and the hydatid cyst layers had calcium deposits, and in the 80 Gy group the hydatid cyst cuticles collapsed and formed calcified lesions and the cuticle fractured.

## 4. Discussion

During surgery to remove hydatid cysts, cyst fluid spillover may cause protoscoleces to attach and the echinococcosis recurs. Drug therapy is generally used to try and kill protoscoleces to prevent this from happening but is often ineffective [6, 7]. The aim of this study was to see if irradiation of cultured protoscoleces using *γ*-rays could prevent echinococcosis in mice when the protoscoleces were implanted into the abdominal cavity, as a model for the human surgical situation. The results showed that the incidence of echinococcosis was decreased with irradiation of protoscoleces.

Previous studies have investigated different methods of preventing recurrent echinococcosis after surgical resection of hydatid cysts; these include drug therapy with recommended drugs such as albendazole [16] and other methods such as inhibition of larval growth with essential oils [17], the use of high-intensity focused ultrasound alone [18] and in combination with a superabsorbent polymer [19, 20], and the use of fungal chitosan [21]. The results of this study suggest that irradiation with *γ*-rays is another method that should be seriously considered as at low doses it was at least as effective as the other methods tested. Irradiation has previously been used to treat hydatid cysts that cannot be removed surgically [22], but irradiation is apparently more successful at killing protoscoleces* in vitro* than* in vivo* [15]. In this study, after abdominal cavity implantation of *γ*-ray irradiated protoscoleces in mice,* Echinococcus* were found in the control group, while the incidence of* Echinococcus* in the experimental groups reduced with increased irradiation dose. This result indicated that the DNA damage in proscolex cells caused by these doses of *γ*-rays was repairable because there was* Echinococcus* formation in the experimental groups. However, with increased exposure dose, the ability to repair appears to decrease, and the incidence of the* Echinococcus* reduces, suggesting a dose-effect relationship between the exposure dose and incidence. In the exposure dose range of 10 Gy to 20 Gy, the weight of the* Echinococcus* produced by protoscoleces reduced with increasing exposure dose, indicating that the structural damage to DNA in the cells of a protoscolex may lead to its biological dysfunction and result in different degrees of* Echinococcus* development. However, in the range of 20 Gy to 80 Gy, the increase of exposure dose no longer induced variations in* Echinococcus *weight. Combined with the variation of incidence of* Echinococcus*, we may consider that in the early irradiation with different doses, the common effect of the damage of protoscolex cells DNA and the body's immune reaction leads to different incidence of the* Echinococcus*. However, once the* Echinococcus* is generated, the body's immune reaction no longer impacts on the* Echinococcus*, which is consistent with Smyth and Heath's opinion [23]. Our subsequent studies found that though the irradiated protoscolex can still generate* Echinococcus*, the effects of the *γ*-ray are sustained, and its targets are mostly focused on the germinal layer of the generated* Echinococcus*. The germinal layer is the material basis of the cuticle layer, while the cuticle layer in turn plays a protective role in the structural function of the germinal layer. In HE staining results, we found that part of the cuticle layers in the experimental groups appeared to show nonuniform growth, as well as detachment, collapse, and even fracture of the fibrous layer, indicating that the normal physiological functions of the germinal layer of the* Echinococcus* generated by an irradiated protoscolex were damaged. Alizarin red staining results showed that* Echinococcus* in the experimental groups contained a large amount of calcification, indicating that the necrotic calcification process of the* Echinococcus* generated by an irradiated protoscolex was accelerated.

This study has some limitations, as the results are preliminary; the mechanisms involved in protoscoleces damage were not investigated. Using a mouse model to investigate the implantation of irradiated protoscoleces into the abdominal cavity may not give the same results as preoperative irradiation in a clinical situation, so these results need to be investigated further in other models and eventually in a clinical study in humans.

## 5. Conclusion

In conclusion, we believe that irradiation with *γ*-rays can kill protoscoleces [14] and reduce the incidence of echinococcosis. Its effects can reduce the generation of* Echinococcus*. If this function of *γ*-rays can be applied to laparoscopic treatment of echinococcosis or other preoperative treatments, postoperative recurrence caused by implantation of echinococcosis may be significantly reduced. However, the full details of any effective method of *γ*-ray therapy remain to be investigated in further studies.

## Figures and Tables

**Table 1:** Analysis of the incidence of cysts in each experimental group.

Group	Number of inoculated mice with cysts	Total number of inoculated mice	Incidence of cysts (%)	*P* value^*∗*^
Control	15	15	100.0	0.005
10 Gy	12	15	80.0	
20 Gy	5	15	33.3	
40 Gy	5	15	33.3	
80 Gy	4	15	26.7	

*Note*. ^*∗*^Chi-square test.

**Table 2:** Cysts weight and suppression capsule rate.

Group	*n*	Median (mg)	Quartile (mg)	Mean (mg)^Δ^	Suppression capsule rate (%)
Control	15	157.80	0.15	15.90	—
10 Gy	15	35.80^*∗*^	0.07	4.20	73.55
20 Gy	15	0.00^*∗*^	0.08	1.92	87.92
40 Gy	15	0.00^*∗*^	0.09	1.92	91.48
80 Gy	15	0.00^*∗*#^	0.00	0.06	99.61

*Note*. Suppression capsule rate (%) = [1 − (mean weight of cyst in the experimental group/mean weight of cyst in the control group)] × 100%. IQR: interquartile range; ^*∗*^ *P* < 0.005 (0.05/10, adjusted *P* value) *versus* control group; ^#^ *P* < 0.005 (0.05/10, adjusted *P* value) *versus* 10 Gy.

Δ: mean cysts' weight calculated according to the median.
